# West Nile Virus in Slovenia

**DOI:** 10.3390/v12070720

**Published:** 2020-07-03

**Authors:** Nataša Knap, Miša Korva, Vladimir Ivović, Katja Kalan, Mateja Jelovšek, Martin Sagadin, Samo Zakotnik, Katja Strašek Smrdel, Jan Slunečko, Tatjana Avšič-Županc

**Affiliations:** 1 Institute of Microbiology and Immunology, Faculty of Medicine, University of Ljubljana, 1000 Ljubljana, Slovenia; natasa.knap@mf.uni-lj.si (N.K.); misa.korva@mf.uni-lj.si (M.K.); mateja.jelovsek@mf.uni-lj.si (M.J.); martin.sagadin@mf.uni-lj.si (M.S.); samo.zakotnik@mf.uni-lj.si (S.Z.); katja.strasek@mf.uni-lj.si (K.S.S.); jan.slunecko@gmail.com (J.S.); 2 Department of Biodiversity, Faculty of Mathematics, Natural Sciences and Information Technologies, University of Primorska, 6000 Koper, Slovenia; vladimir.ivovic@famnit.upr.si (V.I.); katja.kalan@gmail.com (K.K.)

**Keywords:** WNV, mosquitoes, Slovenia, WNND

## Abstract

West Nile virus (WNV) is a flavivirus transmitted by mosquitoes. Birds are the reservoir for the virus; humans, horses and other mammals are dead-end hosts. Infections caused by WNV in humans can vary from asymptomatic infections to West Nile fever (WNF) or West Nile neuroinvasive disease (WNND). In 1995, a serosurvey was performed in Slovenia on forest workers, and WNV specific IgG antibodies were confirmed in 6.8% of the screened samples, indicating that WNV is circulating in Slovenia. No human disease cases were detected in Slovenia until 2013, when the first case of WNV infection was confirmed in a retrospective study in a 79-year old man with meningitis. In 2018, three patients with WNND were confirmed by laboratory tests, with detection of IgM antibodies in the cerebrospinal fluid of the patients. In one of the patients, WNV RNA was detected in the urine sample. In 2017, 2018 and 2019, a mosquito study was performed in Slovenia. Mosquitoes were sampled on 14 control locations and 35 additional locations in 2019. No WNV was detected in mosquitoes in 2017 and 2019, but we confirmed the virus in a pool of *Culex* sp. mosquitoes in 2018. The virus was successfully isolated, and complete genome sequence was acquired. The whole genome of the WNV was also sequenced from the patient’s urine sample. The whole genome sequences of the WNV virus detected in Slovenian patient and mosquito indicate the virus most likely spread from the north, because of the geographic proximity and because the sequences cluster with the Austrian and Hungarian sequences. A sentinel study was performed on dog sera samples, and we were able to confirm IgG antibodies in 1.8% and 4.3% of the samples in 2017 and 2018, respectively. Though Slovenia is not a highly endemic country for WNV, we have established that the virus circulates in Slovenia.

## 1. Introduction

West Nile virus (WNV) is a flavivirus transmitted by mosquitoes. Birds are reservoir for the virus; humans, horses and other mammals are dead-end hosts and do not contribute to circulation and further spread of the virus in nature. The main vector of WNV in Europe are the *Culex pipiens* mosquitoes [1]. Infections caused by WNV in humans can vary in the presentation, from asymptomatic infections to West Nile fever (WNF) or West Nile neuroinvasive disease (WNND) [2].

The first outbreak, caused by WNV in Europe was reported in Romania in 1996, and since then, cases have been reported in several European countries: Albania, Bosnia, Bulgaria, Croatia, Cyprus, France, Greece, Germany, Hungary, Italy, Kosovo, Montenegro, North Macedonia, Portugal, Romania, Russia, Serbia, Spain, Turkey and Ukraine [3,4]. Entomological and veterinary surveillance confirmed that WNV is actively circulating in the countries reporting cases, including countries neighboring Slovenia: Italy, Austria, Croatia and Hungary [5]. Already in 2004, the virus was isolated and confirmed WNV lineage 2 in Hungary and by 2008 in Austria [6,7]. The virus spread further through the Balkan countries, and by 2011, first cases caused by WNV lineage 2 were detected in Italy. In Austria, the first cases were confirmed retrospectively already in 2009 and 2010, and cases of WNV in humans have appeared annually since 2014 [8]. Human neuroinvasive WNV infections were first documented in Hungary in 2003 already, and since then, 15 to 20 cases have been regularly diagnosed [9]. In Croatia, first 7 human cases of WNND were confirmed in 2012, with sporadic cases having been reported annually since then and smaller outbreaks reported in 2013 and 2017 [10]. In Italy, the virus confirmed in 2008, with WNV lineage 1 causing human WNND [11]. In 2011 WNV lineage 2 was confirmed in Italy, and since then, the number of cases has risen significantly [11]. After first human cases were detected in the neighboring countries, mosquito surveillance was introduced, namely by Italy in 2009, Hungary in 2010, Austria in 2011 and Croatia in 2012 [8,9,10,11], which often served as the support for the early-warning systems and confirmed WNV presence in the countries. 

For almost a decade after the first evidence of virus circulation in neighboring countries, no human cases were diagnosed in Slovenia. Only in 2013, the first human case was confirmed in a retrospective study.

The goal of the study was to detect the local virus circulation in Slovenia by analyzing the presence of WNV in mosquitoes in Slovenia and by establishing the presence of the diseases in patients in Slovenia. A study of mosquito populations was performed in the past three years to evaluate the WNV presence as well as retrospective analysis of human meningitis cases. Additionally, to aid this objective, a WNV serosurvey was performed on dog sera samples to estimate their potential role as sentinels. The data obtained in these studies are described in this article with the aim to explain the virus circulation in Slovenia.

## 2. Materials and Methods

### 2.1. Mosquito Trapping

**Figure 1:**
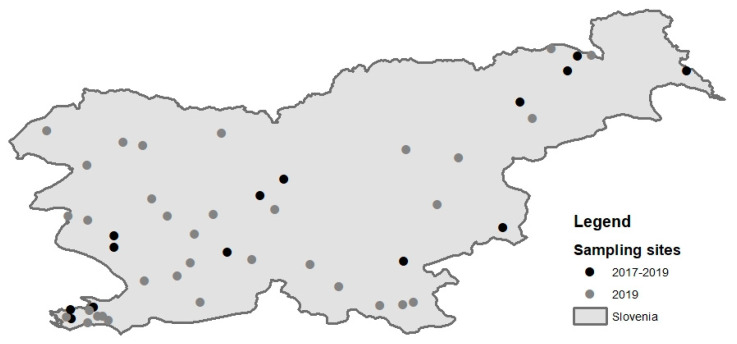
Mosquito sampling locations in Slovenia. Black dots represent sampling locations in years 2017–2019; grey dots represent additional locations sampled in 2019.

From July 2017 to October 2019, mosquitoes were sampled throughout Slovenia (Figure 1). We sampled mosquitoes in 14 locations in Slovenia, to ensure surveillance of a significant part of the country, and in 2019 we introduced 35 additional locations. The mosquitoes were sampled using BG-Sentinel traps and CDC traps baited with CO2, which operated for 24 h, thereby enabling the capture of diurnal and nocturnal species of mosquitoes. The traps were set in rural and urban areas in the vicinity of water sources or close to animals. Captured mosquitoes were collected, put on dry ice in the field and maintained under cold conditions throughout the testing process. The identification of adults was done on a chill plate and was based on available keys [12,13,14]. With few exceptions, where collected mosquitoes were screened for viruses without identification, the mosquitoes were identified to the species level, counted and pooled according to date and location. The mosquitoes were tested for flavivirus presence in pools up to 30 animals. A total of 179 mosquitoes, grouped in 40 pools; 3054 mosquitoes, grouped in 246 pools and 7900 mosquitoes, grouped in 1009 pools, were screened for WNV in 2017, 2018 and 2019 respectively. Each pool of mosquitoes was homogenized in 600 µL of RPMI Medium using Tissue Lyser (Retsch for Qiagen, Hilden, Germany). Two hundred µL of the homogenate was stored for future analysis; 200 µL was inoculated into cell cultures (Vero E6 and C6/36), and 200 µL was used for molecular analysis.

### 2.2. Virus Detection

Nucleic acid was extracted from the aliquotes of mosquito homogenate and human whole blood samples with the BioRobot EZ1-XL Advanced (Qiagen) using the EZ1 Virus Mini Kit v2.0 (Qiagen) and eluted in 60 µL. For RNA extraction from human urine samples MagNA Pure Compact System MagNA Pure Compact Nucleic Acid Isolation Kit I-Large Volume was used. WNV was detected by real-time RT-PCR [15]. Briefly, the PCR was performed on Applied Biosystems 7500 Fast Real-Time PCR System. The reactions were carried out in a total volume of 20 µL, containing 5 µL of RNA, 5 µL of TaqMan^®^Fast Virus 1-Step Master Mix (Applied Biosystems, Thermo Fisher Scientific, Grand Island, NY, USA), 1 µM of each primer and 0.3 µM of probe. Cycling conditions were as follows: 95 °C for 20 s, 40 cycles of 95 °C for 15 s and 60 °C for 30 s. The prevalence of infection was calculated using the program PooledInfRate version 3.0 (a Microsoft^®^ Excel Add-In, developed by Brad Biggerstaff, CDC, Fort Collins, CO, USA).

### 2.3. Case Laboratory Investigations

Possible cases of WNND and WNF occurring in Slovenia were referred to the Institute of Microbiology and Immunology, Faculty of Medicine, University of Ljubljana. Molecular diagnostics was performed on patients’ whole blood, urine and cerebrospinal fluid samples with real-time RT-PCR as described above. Detection of WNV IgM and IgG antibodies in serum and cerebrospinal fluid samples was done by ELISA (WNV IgM capture DxSelect ELISA and IgG DxSelect ELISA kits, Focus Diagnostics, Cypress, CA, USA).

### 2.4. Retrospective Surveillance of Patients with Meningitis/Meningoencephalitis

Since 2011, we have retrospectively investigated WNV infection in all patients with meningitis/meningoencephalitis that were TBEV IgM and IgG negative or were only IgG positive (due to previous TBE vaccination or past in apparent TBE infection) and were >65 years of age. Enzygnost Anti-TBE/FSME Virus assay (Siemens, Marburg, Germany) was used for detection of TBEV IgM and IgG antibodies. The stored samples were tested at the end of season for the presence of WNV IgM and IgG antibodies in serum, and cerebrospinal fluid samples were done by ELISA (WNV IgM capture DxSelect ELISA and IgG DxSelect ELISA kits, Focus Diagnostics, Cypress, CA, USA).

### 2.5. Screening of Dog Sera

Dog sera were referred to the Institute of Microbiology and Immunology for antibody screening against *Anaplasma phagocytophilum*. The samples were sent from different regions in Slovenia. For screening sera were diluted 1:40 and tested using indirect immunofluorescence assay on spot slides containing Vero E6 cells infected with WNV (strain: Eg101). As conjugate anti-Dog IgG-FITC (No. F7884, Sigma-Aldrich, St. Louis, MO, USA) in the dilution 1:32 was used. The sera from seropositive dogs were further diluted to establish the end-point titer.

### 2.6. Virus Neutralization Assay

To confirm the presence of WNV specific neutralizing antibodies in dog sera we performed virus neutralization assay. Two-fold serial dilutions (starting dilution was 1:10) were mixed with 100 TCID50 of WNV (strain: Eg101) and incubated for 1 h at 37 °C. Subsequently, 50 µL virus-antibody mix were inoculated on 96-well plate containing 80% confluent Vero E6 monolayer. After 1.5 h 50 µL of fresh DMEM containing 4% fetal bovine serum (FBS) was added to each well. Plates were incubated for 4 days at 37 °C, before they were fixed with 50 µL of 4% formaldehyde. Afterwards, plates were checked for cytopathic effect (CPE) under the microscope, and cell monolayer was additionally visualized using crystal-violet staining. The antibody titer was defined as the reciprocal of the highest dilution of the serum that showed 100% neutralization. Positive and negative control sera were included in each plate. Sera with a titer of 1:20 were considered positive.

### 2.7. Virus Isolation

Each 100 µL pool aliquot of mosquitoes was inoculated onto 6-well plate containing 80% confluent monolayer of Vero E6 or C6/36 cells. Briefly, after inoculation, Vero E6 cells were incubated 1 h at 37 °C; then, 2 mL of fresh DMEM containing 4% FBS was added, and plates were incubated at 37 °C in a 5% CO2 atmosphere for 7 days. C6/36 cells were first incubated for 1 h at room temperature, and subsequently, 2 mL of L-15 media containing 10% FBS was added to each well, and plates were further on incubated for 7 days at 28 °C. All plates were daily examined for presence of CPE. The cells were blindly passaged 3 times.

Additionally, the homogenate of the mosquito pool Ko169/19, where WNV RNA was detected, was inoculated intracerebrally into suckling mice BALB/c. Six days after infection, 1 of the 4 infected mice showed symptoms of infection (loss of balance). The mice were euthanized with CO2, and brains were removed. WNV RNA was detected in the brain of the infected rodent, and the tissue was inoculated on Vero E6 cells as described above.

### 2.8. Complete Genome Sequencing

For sequencing, RNA was isolated with Direct-zol RNA Kit (Zymo Research, Irvine, CA, USA), following manufacturer’s instructions. From the mosquito sample, Ko169/19 cDNA was transcribed with Maxima H Minus Double-Stranded cDNA Synthesis Kit (Thermo Scientific) and further amplified using SISPA protocol to acquire sufficient quantities. For construction of NGS library, we used 2 different Nextera kits (Nextera Flex kit with Nextera DNA CD Indexes and Nextera XT kit with Nextera XT Index Kit, Illumina, San Diego, CA, USA), according to manufacturer’s instructions (Illumina, San Diego, CA, USA). From the human sample, WNV genome was reverse transcribed and amplified using overlapping genome specific primers kindly provided by Victor Corman (Charité-Universitätsmedizin Berlin, Germany). PCR amplicons ranging from 486 to 806 bp were excised from UltraPure™ Agarose gel (Invitrogen, Life Tehnologies, Carlsbad, CA, USA) and purified using QIAEX II Gel Extraction Kit (Qiagen). Concentrations of PCR amplicons were then determined with Qubit™ dsDNA HS Assay Kit (Invitrogen, Life Tehnologies, Carlsbad, CA, USA). For preparation of NGS library, equimolar concentrations of all 20 PCR amplicons were prepared, and NGS library was constructed with Nextera XT kit and labelled with Nextera XT Index Kit according to manufacturer’s instructions (Illumina, San Diego, CA, USA). All NGS libraries were sequenced with Miseq Reagent Kit V3 on a MiSeq system (Illumina, San Diego, CA, USA) in two separate runs.

### 2.9. Phylogenetic Analysis

Data from the first run was filtered, and adapter sequences were removed with Trimmomatic (default settings, Illuminaclip was performed with inbuilt adapter sequences for Nextera pair end libraries) [16], and genome was de novo assembled using Unicycler (default settings) on Galaxy server [17]. Data from the second run was filtered, and adapter sequences were removed with BBduk (settings: qtrim = rl, trimq = 10, maq = 10, entropy 0.5, entropywindow = 50, entropyk = 5 and mapping to inbuilt adapter sequences for Nextera libraries). First library yielded 8.0 × 105 reads (paired at 2 × 301 nt) and second yielded more than 1.7 × 106 reads. The amplicon sequencing from patient’s sample yielded 1.3 × 105 reads. Obtained data were processed and mapped on WNV reference genome (KM659876) using Bowtie2 (default settings) [18]. We then used BFC tools [19] to extract the WNV sequence. WNV sequence was further optimized using PILON (default settings) [20]. The newly obtained genomes were deposited in the GenBank under the accession numbers: MK947396 and MK947397. Phylogenetic analysis was performed on all full length WNV genomes available in GenBank on 25.3.2019 using muscle. The IQ-TREE [21] was used to perform phylogenetic analysis under the GTR+F+R2 model as best predicted model using the ultrafast bootstrap option and to draw a phylogenetic tree of 26 most closely related strains.

### 2.10. Ethics Statement

Human and animal samples were sent to the Institute of Microbiology and Immunology, Faculty of Medicine for diagnostic purposes. No samples were specifically collected for this study. All procedures involving animals were approved by the National Ethical Committee and the Administration of the Republic of Slovenia for Food Safety, Veterinary and Plant Protection (Permit number 34401-7-2016-5, 31 January 2017). Animal care and treatment were conducted in accordance with the institutional guidelines and international laws and policies (Directive 2010/63/EU on the protection of animals used for scientific purposes).

## 3. Results

### 3.1. Mosquito Trapping and Virus Detection

**Figure 2:**
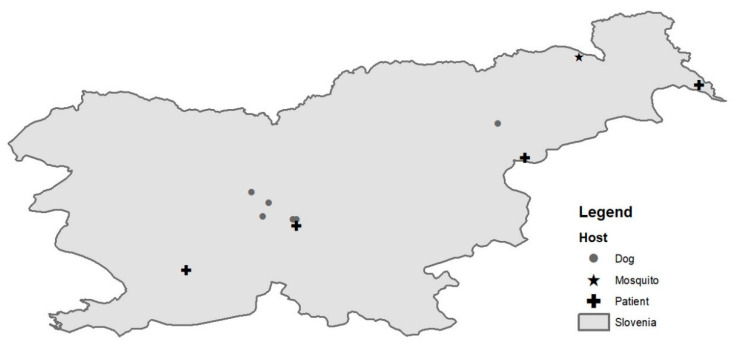
A map of geographic distribution of West Nile virus (WNV) positive patients (cross), mosquito pool (star) and dogs (point) identified in Slovenia in 2018.

A total of 199, 3054 and 7900 mosquitoes were captured in Slovenia in 2017, 2018 and 2019, respectively, at 14 locations in 2017 and 2018 and 35 additional locations in 2019 (Figure 1). The captured mosquitoes belonged to the following genera: *Aedes*, *Ochlerotatus*, *Culex*, *Anopheles*, *Culiseta* and *Coquillettidia*. The species distribution is described in Table 1. They were organized in 1295 pools (40 in 2017, 246 in 2018 and 1009 in 2019). We detected WNV RNA (ct value 20.0) in a single pool of 3 *Culex pipiens* mosquitoes captured in August 2018 in a village in north-eastern Slovenia, close to the Austrian border (Figure 2).

### 3.2. Case Laboratory Investigations

During the surveillance period in 2018, three patients, 2 males and 1 female, aged 59–70 years, of 113 possible WNND were confirmed by laboratory tests (all WNND cases were IgM and IgG-positive, confirmed by neutralization test, while WNV RNA was detected in 1 urine sample).

### 3.3. Retrospective Surveillance of Patients with Meningitis/Meningoencephalitis

Table 2 presents the results of the retrospective study. We confirmed antibodies against WNV in one sample in 2013 and thereby confirmed the first case of WNND case in Slovenia. In 2018 we found two additional acute WNV cases, one of them presented WNND. Since TBEV, which is endemic in Slovenia, produces cross-reactive antibodies to WNV, all of the samples showing antibody reactivity to TBEV were excluded as WNV infections. All the confirmed WNV cases were reported to the ECDC [22].

### 3.4. Screening of Dog Sera

Dog sera samples were screened for the presence of WNV specific antibodies. In 2017, we detected antibodies in 3 out of 209 sera samples (1.4%), and in 2018, we confirmed WNV infection in 8 out of 216 (3.7%) tested dogs. All dog sera had neutralizing antibodies with titers ranging from 1:40 to 1:320. 

### 3.5. Genome Sequencing and Phylogenetic Analysis

**Figure 3:**
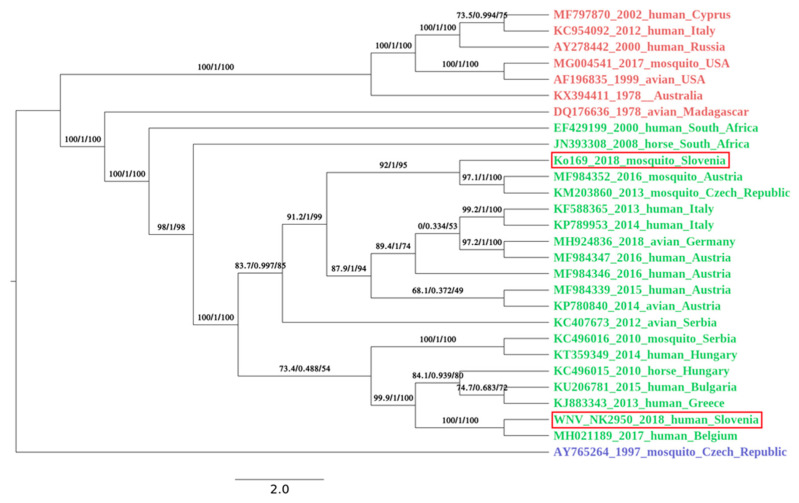
A phylogenetic analysis was performed with IQ-TREE using ultrafast bootstrap approximation [23] under the GTR+F+R2 model (chosen by ModelFinder [24]) on all available full length WNV genomes available in the GenBank on the 25 March 2019. For better visualization of a phylogenetical relatedness of Slovenian WNV strains, a subset of 28 genomes, with emphasis on geographic closeness of the origin countries, was chosen for Figure. Lineage are color coded: red, lineage 1; green, lineage 2 and blue, lineage 3. Both Slovenian isolates (circled in red) belong to the virus lineage 2. The mosquito pool (Ko169/18; GenBank no. MK947396) is phylogenetically closer to the strains circulating in Austria and Czech Republic. The human isolate (NK2950; GenBank no. MK947397) is most closely related to the WNV strain circulating in Hungary in 2017. The first value on the branches represents SH-like approximate likelihood ratio test result in % [25]; second value approximates Bayes test result in % [26], and third value is ultrafast bootstrap approximation [27]. The bar in the legend represents the number of nucleotide substitutions per site alongside the branches.

Complete genome sequence was successfully obtained from mosquito pool and a patient’s urine sample. From first library, prepared from mosquito pool, we were able to de-novo assemble the complete WNV sequence, and the data from the second run was used to generate better coverage. The complete genome sequence was 10,317 bp long and had one open reading frame (5–10,309 nt) translating the genome into one polyprotein. The average coverage was 7.3. Since WNV RNA in a patient’s urine sample was detected at very low concentration (ct value of 29.7), we decided to use amplicon sequencing to obtain complete WNV cds. The complete sequence was 11,017 bp long, and the average coverage was 6846. Nucleotide alignment of Slovenian WNV sequences showed 92.9% identity on the nucleotide level. Both newly obtained Slovenian WNV sequences belonged to the Lineage 2 and clustered well within the sequences obtained in the neighboring countries. The WNV sequence obtained from the first acute Slovenian patient (NK2950; GenBank no. MK947397) was most closely related (99.6% on the nt level) to the WNV sequence obtained from Belgian patient that contracted WNV in Hungary in 2017 (Figure 3). The patient is the resident of the central part of Slovenia (Figure 2). On the other hand, the mosquito pool (Ko169/18; GenBank no. MK947396) originated from north-eastern Slovenia (Figure 2) and was phylogenetically closer to the strains found in Austria (99.7% identity on the nt level), Czech Republic (99.8%) and Italy (99.6%) (Figure 3).

## 4. Discussion

Slovenia is a very heterogenous country located on the transient area between the Balkans and Central Europe. The influence of the Mediterranean and the Alps is seen in the country and affects both fauna and flora. Slovenia is a stopover for migratory birds, and therefore, we see significant biodiversity in bird population [28]. In Slovenia serosurvey of wild passerine birds during autumnal migration was performed from 2004 to 2009, and 4.7% of captured birds were found to harbor WNV specific IgG antibodies [29]. During the same period, a survey was performed on country poultry, and IgG antibodies were confirmed in two pheasants, confirming circulation of the virus in Slovenia already in that period [30]. The knowledge on Slovenian mosquito populations and their vector potential is limited. The first pilot mosquito monitoring was performed in 2012 [31]. Additionally, studies on invasive mosquito species were carried out in the following years [32,33]. Only one study assessed the presence of pathogens in mosquito populations in Slovenia, and until now, only mosquito-only flaviviruses were confirmed [31]. Previous studies were done on a limited number of locations in Slovenia or implemented capture methods, which focused on invasive species. This is the first study where capture of all mosquito species was targeted on a wider geographic area.

One hundred ninety-nine, 3054 and 7009 mosquitoes were tested in 2017, 2018 and 2019, respectively. No WNV RNA was detected in mosquito pools in 2017 and 2019, but we confirmed WNV RNA in a single pool of *Culex pipiens* mosquitoes captured in August 2018. The minimum infection rate, 2,4 × 10^−4^, was significantly lower compared to those detected in neighboring Austria (0,39) in non-epidemic years [8], indicating a lower potential for viral emergence. The sampling region where the mosquito pool was captured is close to the Austrian border. The virus was isolated and fully sequenced and belongs to the WNV Lineage 2. Phylogenetically, it is closely related to strains found in mosquitoes in Austria and the Czech Republic. The aim of our study was not entomological surveillance, but such a system would have been able to detect WNV circulation before the human cases arise, as it is seen in Italy [11]. Thus, our study highlights the importance of early warning systems in WNV circulation. However, due to the complexity of the WNV enzootic cycle, an interdisciplinary approach is necessary for the surveillance of WNV circulation. Entomological, veterinary and human surveillance systems should be integrated for setting up timely preventive measures.

In 1995, a serosurvey study of various arboviruses was performed on sera samples obtained from forest workers in Slovenia in order to estimate the extent of exposure of these viruses in population at risk. WNV specific IgG antibodies were confirmed in 6.8% of the screened samples indicating that WNV is circulating in Slovenia [34]. The first human case of WNV infection in Slovenia was confirmed retrospectively in 2013 in a 79-year old man, who was hospitalized with meningitis [35]. From 2014 to 2017, several European countries reported locally acquired WNF cases, but no cases were confirmed in Slovenia. Most acquired cases were reported between July and October, with case numbers peaking between mid-August and mid-September [36]. In 2018, the first cases were notified by Greece already at the end of June indicating a usually early start of the WNV transmission season [36]. In Slovenia, we detected the first case of acute WNV infection in August 2018. Two more cases were detected in August and September (ECDC). In 2018, two additional WNND cases were confirmed retrospectively, one of them probably an imported case and the other a locally acquired case from July 2018, which was earlier than the detection of WNV RNA in mosquitoes or detection of the first WNND human case. WNND was not considered for the patient diagnostic panel, since there was no evidence of active WNV transmission in Slovenia at the time, and no human disease cases were detected in Slovenia before August 2018, even though virus presence had been established decades ago. With the emergence of Lineage 2 in Europe in 2004, the burden of WNV in Europe has changed significantly. Though there is significant interannual variation in the incidence of WNV in Europe, there have been several peak years, with the most pronounced ones in 2013 and 2018 [37]. In 2018, we saw the largest increase in the number of human cases in Europe, and the elevated temperatures in the summer and an early spring have been associated with WNV epidemics in Europe [38]. Additionally to the numerous human cases, the virus was detected for the first time in birds in Germany, and a high mortality rate of raptors was reported in the Czech Republic [39,40], indicating the virus spread in Europe as well as the intensity of the epidemics. The whole genome sequences of the WNV detected in the Slovenian patient and mosquitos indicate the virus most likely spread from the north, because of the geographic proximity and because the sequences cluster with the Austrian and Hungarian sequences (Figure 2). Nevertheless, there are indications that the virus was present in Slovenia in the years prior to 2018. A sentinel study performed on dog sera collected in Slovenia indicated the presence of WNV specific antibodies already in 2017. The number of dogs with specific WNV antibodies was considerably higher in 2018, indicating a more intensive transmission of the virus. In addition, the dogs with WNV antibodies were confirmed in the same regions where we were able to detect WNV infections in humans, and the virus was confirmed in birds and a horse [41].

WNV was detected in Slovenia decades ago, but the virus caused no notable disease in humans, birds or horses. With the introduction of a new lineage to Europe and the changes that we see in our environment, the transmission of the virus has become increasingly likely, especially in years when the climate supports the increase in the abundance of mosquitoes. Therefore, monitoring of mosquitoes as well as the knowledge of clinicians and public health officials regarding the virus’ presence in our environment are necessary to introduce the preventive measures, for example screening of blood donations and real-time diagnosis of patients.

## Figures and Tables

**Table 1:** Mosquito species distribution in the three years of the study.

Mosquito Species	2017	2018	2019
*Culex* sp.	54.75%	96.04%	25.01%
*Aedes/Ochlerotatus* sp.	21.79%	1.34%	68.98%
*Anopheles* sp.	20.11%	1.98%	3.05%
*Coquillettidia* sp.	2.79%	0.35%	1.29%
*Culiseta* sp.	0.56%	0.29%	1.67%

**Table 2:** Retrospective testing of TBEV negative meningitis/meningoencephalitis cases, older than 60 years.

Year	Number of Patients Examined *	Period of the Year	ELISA IgG—WNV Reactive	ELISA IgM—WNV Reactive	ELISA—IgG TBE Reactive and ELISA—IgG WNV Reactive
2011	44	August–September	4	0	4 out of 4
2012	35	August–September	0	0	0
2013	27	August–September	3	1	2 out of 3
2014	145	January–December	10	0	6 out of 10
2015	102	January–December	2	0	2 out of 2
2016	98	June–September	6	0	6 out of 6
2017	84	June–September	0	0	0
2018	126	June–September	4	2	2 out of 4

* patients with meningitis/meningoencephalitis that were TBEV IgM TBEV IgM and IgG negative or were only IgG positive.
